# The therapeutic effectiveness of oral cannabidiol in addition to current treatment in cats with osteoarthritis

**DOI:** 10.18849/ve.v9i4.691

**Published:** 2024-10-23

**Authors:** Oliver Wilkinson

**Affiliations:** University of Bristol, United Kingdom

**Keywords:** cannabinoid, cannabidoil, cat, cbd, feline, locomotion, oa, osteoarthritis, pain

## Abstract

**PICO question:**

In cats with osteoarthritis (OA), does the oral supplementation of cannabidiol (CBD) oil, compared to conventional treatment alone, improve treatment outcomes of reducing pain and improving locomotion?

**Clinical bottom line:**

**Category of research:**

Treatment.

**Number and type of study designs reviewed:**

No papers were found relevant to the PICO.

**Strength of evidence:**

Zero.

**Outcomes reported:**

No studies relevant to the PICO were found (in English) on the use of CBD in cats with OA.

**Conclusion:**

There is currently no evidence to recommend the use of CBD in cats for the treatment of OA and further studies on therapeutic use of CBD in cats with OA are required.

## How to apply this evidence in practice

The application of evidence into practice should take into account multiple factors, not limited to: individual clinical expertise, patient’s circumstances and owners’ values, country, location or clinic where you work, the individual case in front of you, the availability of therapies and resources.

Knowledge Summaries are a resource to help reinforce or inform decision-making. They do not override the responsibility or judgement of the practitioner to do what is best for the animal in their care.

## Clinical scenario

An owner presents a 10-year-old cat with diagnosed osteoarthritis (OA) causing chronic pain, which is on long-term NSAIDs for analgesia. However, the cat is still showing signs of discomfort. The owner is unable to afford monoclinal antibody treatment (frunevetmab) and enquires about the effectiveness of cannabidiol (CBD) as an additional supplement.

## The evidence

There is no peer-reviewed evidence in cats that meets the inclusion criteria of the PICO.

## Appraisal, application and reflection

OA is a common condition seen in older companion animals, with numerous useful first-line treatments in both cats and dogs. However, none of these are 100% risk-free and those with less risk are also expensive in the long term. There has been a lot of research published in recent years into the use of cannabis and CBD in humans for analgesia but a very limited amount in dogs, and none in cats.

Of the papers found, only one paper presented any data regarding cats (Milevoj et al., 2022). However, the data set does not relate to the PICO, as while there are results regarding cats, none are related to OA. The results were also generated by owner surveys so therefore are likely to be subjective, being based on owner views, and, with only 4 responses in cats, the results cannot be considered representative or exhaustive.

A recent case study (Gutierre et al., 2023) suggested that CBD could be used as a therapeutic alternative for cats with OA. However, the CBD mixture contained 0.8% THC (Tetrahydrocannabinol), so cannot be compared to other studies (and is impossible to replicate legally in multiple countries due to controlled drug regulations). A solo case study provides poor evidence of the effectiveness of CBD. In addition, the cat was also on prednisolone and had several concurrent health conditions, so would not have been a good candidate for study into the effectiveness of CBD.

The other studies identified, relating to CBD use in cats, were focusing on safe dosage levels and potential side effects, but none investigated therapeutic outcomes (Kulpa et al., 2021; Wang et al., 2022).

There appears to be weak evidence for CBD being effective in dogs with OA (Gamble et al., 2018; Verrico et al., 2020; Mejia et al., 2021). These papers conclude that more robust studies with increased sample sizes are needed to provide sufficient evidence as to the effectiveness of CBD in OA in either species. The most recent study (Patikorn et al., 2023) also concluded that CBD is considered safe to use in dogs and may reduce pain scores but to be certain more high-quality randomised controlled studies are needed.

Eight papers were identified by a database search, three were review articles, and the other five were not relevant. Most papers focused on dogs and only two papers had any data pertaining to cats which was not relevant to the PICO. Overall, none of the papers qualified for inclusion.

The author did a broader search based on the strategy below (including only species and intervention) to ensure no papers were missed. This resulted in 54 papers; however, all were screened out under exclusion categories listed.

## Methodology

**Search strategy:** 

Databases searched and dates covered:	CAB Abstracts on Ovid platform from 1973 to September 2023 Ovid MEDLINE on Ovid platform from 1946 to September 2023
Search terms:	CAB Abstracts: exp cats/ (cat or cats or feline*).tw. 1 or 2 osteoarthritis/ (osteoarthritis or osteoarthritides).tw. 4 or 5 exp cannabinoids/ (CBD or cannabidiol or cannabinoid*).tw. 7 or 8 3 and 6 and 9 OVID Medline: Cats/ (cat or cats of feline*).tw. 1 or 2 Osteoarthritis/ osteoarthritis or osteoarthritides).tw. 4 or 5 cannabinoids/ or cannabidiol/ (CBD or cannabidiol or cannabinoid*).tw. 7 or 8 3 and 6 and 9
Dates searches performed:	21 Sep 2023

Table note placeholder

**Exclusion / inclusion criteria:** 

Exclusion	Review articles. Non-English papers. Non-relevance to PICO.
Inclusion	Articles covering cats or dogs. Articles using CBD or cannabidiol.

Table note placeholder

**Search outcome:** 

Database	Number of results	Excluded – Non- English	Excluded – Review article	Excluded – Non- relevance	**Total re**levant papers
CAB Abstracts	7	0	2	5	0
PubMed	3	0	3	0	0
Total relevant papers when duplicates removed					0

Table note placeholder

## Acknowledgements

The author wishes to acknowledge Emma Place, Subject Librarian at the University of Bristol, and Ellie Sellers, Clinical Demonstrator, who gave great support and guidance.

## ORCiD

Oliver Wilkinson: https://orcid.org/0009-0006-1992-4694

## Conflict of interest

The author declares no conflicts of interest.

## Disclaimer

Knowledge Summaries are a peer-reviewed article type which aims to answer a clinical question based on the best available current evidence. It does not override the responsibility of the practitioner. Informed decisions should be made by considering such factors as individual clinical expertise and judgement along with patient’s circumstances and owners’ values. Knowledge Summaries are a resource to help inform and any opinions expressed within the Knowledge Summaries are the author’s own and do not necessarily reflect the view of the RCVS Knowledge. Authors are responsible for the accuracy of the content. While the Editor and Publisher believe that all content herein are in accord with current recommendations and practice at the time of publication, they accept no legal responsibility for any errors or omissions, and make no warranty, express or implied, with respect to material contained within. For further information please refer to our Terms of Use.
